# A Qualitative Study of Bottlenecks and Causes of Fractions for Dedicated Incomes of Health Centers and Solutions for their Reduction

**DOI:** 10.5539/gjhs.v8n9p58

**Published:** 2015-10-16

**Authors:** Jafar Sadegh Tabrizi, Saeide Alidoost, Hossein Mashhadi Abdolahi

**Affiliations:** 1 Tabriz Health Services Management Research Center, School of Health Management and Medical Informatics, Tabriz University of Medical Science, Tabriz, Iran; 2 School of Health Management and Medical Informatics, Tabriz University of Medical Science, Tabriz, Iran; 3 Tabriz Health Services Management Research Center, Tabriz University of Medical Science, Tabriz, Iran

**Keywords:** health centers, income, fractions

## Abstract

**Background:**

Primary health care is one of effective approaches for improving public health. Providing optimal cares requires supplication of various resources such as financial resources. “Fractions of incomes” in health centers is one of the remarkable problems for the domain of financial resources management in Iran. This study was aimed to identify bottlenecks and causes of fractions for incomes in health centers and solutions for their reduction.

**Methods:**

The current study was conducted in a qualitative phenomenology method in East Azerbaijan province of Iran in 2014. Data collection method was focus group discussion and semi-structured interview. Purposive sampling was used for selecting participants. Focus group discussions and interviews were conducted based on pre-prepared guidance and continued till data saturation. Validity of guidance was approved by qualitative studies experts. Data were analyzed using content analysis method.

**Results:**

Based on the opinions of participants, two and six themes were respectively extracted for bottlenecks of fractions and causes and solutions for their reduction. Themes for bottlenecks of fractions included cash (monetary) and non-cash (non-monetary) fractions and themes for causes and solutions included causes and solutions for fractions per capita, insurance deductions, fractions related to sending documents, registration fractions, discounts fractions, and incomplete deposit of cash incomes.

**Conclusion:**

All cash and non-cash incomes of health centers are subject to fractions. The causes of fractions are related to the whole process of converting services to incomes and insurance requirements. Identified solutions and interventions also focus on these areas.

## 1. Introduction

The main objective of health organizations is to improve health. One of the approaches of Iran’s health system for health improvement is the establishment of primary health care that has a significant impact on providing health care services and health indicators (Asadi-Lari, Sayyari, Akbari, & Gray, 2003). Provision of health services requires balanced and equitable distribution of many resources especially financial resources.

The main financial resources of health care institutions include government’s budgetary, revenues of institutions and donated funds which institutions’ revenue is more reliable, and delays in the payment of government subsidies and delays in the insurance reimbursement are among the challenges related to financing (Akortsu, & Abor, 2011; Ejughemre, 2014). According to world bank report in 2006, financial difficulties were one of the significant problems at the first level of providing services in Iran and essential interventions were needed to improve financial management of health sector (Abolhallaje, Jafari, Seyedin, & Salehi, 2014; Policy document to promote health care). The costs of health sector cannot be covered by its incomes because of the nature of health centers and providing non-profitable services by them (Stimpson, Li, Shiyanbola, & Jacobson, 2014).

The revenue rate of health sector at medical science universities of Iran has increased within recent years, as health sector share of specific revenue rate was 12% in 2010 (Hasani et al., 2013). Achieving all the expected revenues for the system of health networks and the issue of income fractions for health centers is one of the important components in financial management of health sector. Fraction is defined by monetary (Rial) differences between what should be achieved in return for service provision (based on the approved tariffs) and what is actually achieved. Failure to achieve for all incomes and the applied fractions in health centers lead to loss of financial resources in these centers and thus limit the financial power for the health care networks. As main part of all health care spending is publically financed, public financing constraints may reduce public coverage for some services (OECD/European Union, 2014). The amount of fractions for family physician program in East Azerbaijan were more than 30 billion Rials and accounts for approximately 2% of per capita incomes in 2012 (*Health insurance office. reports of adjustments*). Now if insurance deductions and cash (monetary) fractions would be added to this amount, it will take up a significant share of health center dedicated incomes.

In many studies which have been carried out in relation to hospital fractions, results showed that incomplete documentation records, miscalculation and errors in insurance documents are the major causes of hospitals fractions (Abolhallaje et al., 2013; Askary, Dehghan, Bahrami, & Keshmiri, 2010; Karimi, Vesal, Saeidfar, & Rezayatmand, 2010; Khademolqorani & Zeinal Hamadani, 2015; Tavakoli et al., 2015). But these studies only investigated insurance deductions and other aspects of income fractions have not been studied yet. The present study was an attempt to identify bottlenecks and causes of fractions for incomes in health centers and to provide solutions for their reduction

## 2. Methods

This was a qualitative study carried out in East Azerbaijan province of Iran in 2014. Phenomenology approach was used since the purpose of the current study is to investigate experiences of experts. Participants were sampled purposively (homogenous sampling) from health deputy of Tabriz University of Medical Sciences, health networks and health insurance organization which are governmental and provincial organizations. The inclusion criteria were: having experience in the field of financial resource management, having related education and being interested to participate in the interviews and discussions. Selected participants consisted of income, finance, accounting and budget experts who were experts in financial discussions and fractions in health centers. Focus group discussion and interview methods were used for data collection. For identifying bottlenecks, one focus group discussion and five interviews were carried out. Also, two focus group discussions and nine individual interviews were conducted for studying causes and solutions for the reduction of fractions. Overall, three focus group discussions were formed and the number of participants in these group discussions were 11, 11, and 8 respectively. Four people refused to participate in discussions and interviews. Generally, thirty and fourteen people were respectively selected for discussions and interviews. Focus group discussions were carried out by three of the researchers including one facilitator, one observer, and one scriber. Discussions were conducted with the explanations of the facilitator about objectives of the study, method of performing focus group discussions, fractions and its different types and according to the general framework of pre-designed questions. Discussions were formed in the place for health deputy and each of them lasted for 90-120 minutes. Semi-structured interviews were face to face and conducted by an investigator in participants’ offices and according to the interview guidance. Guide form was prepared according to objectives of study and questions of it were provided in researchers’ manner. Validity of this guidance was approved by qualitative studies experts. Each interview lasted for approximately 60 minutes. Interviews and discussions continued until reaching data saturation, so that they did not lead to any new findings. Permission for voice recording and taking notes was granted by the participants before conducting focus group discussions and interviews.

Sound recordings of group discussions and interviews were carefully transcribed and entries were completely reviewed and readout and were corresponded with the taken notes multiple times. Using content analysis method, transcribes were coded, themes and subthemes were extracted. To increase the reliability of the study, these actions were carried out by two of the researchers. Also, to increase credibility and conformability, the data were sent to the participants of focus group discussions and interviews in writing form and corrected and completed opinions of the participants were applied to the final results.

## 3. Results

From forty-four participants, 30 people participated in focus group discussions (FGD). Most participants were finance and accounting experts. Characteristics of participants have been exhibited in Table 1.

**Table 1:** The characteristic of the study participants

Variable		FGD Number (%)	Interviews Number (%)
gender	Male	23 (77%)	12 (86%)
	Female	7 (23%)	2 (14%)

Median age		38 years	43 years

Education Status	Diploma	4 (13%)	0 (0%)
	Bachelor of Science	21 (70%)	9 (64%)
	Master of Science	2 (7%)	0 (0%)
	Doctorate	3 (10%)	5 (36%)

Professional Experience	Less than 10 years	8 (26%)	1 (7%)
	10-20 years	5 (17%)	9 (64%)
	More than 20 years	17 (57%)	4 (29%)

Profession	Budget Expert	2 (7%)	2 (14%)
	Income Expert	10 (33%)	3 (22%)
	Finance and accounting Expert	14 (47%)	7 (50%)
	Other Experts	4 (13%)	2 (14%)

Affiliated to organizations	Health insurance organization	3 (10%)	2 (14%)
	Health networks	23 (77%)	8 (57%)
	Health deputy	4 (13%)	4 (29%)

Total		30 (100%)	14 (100%)

According to the experiences and opinions of the participants, two themes including cash and non-cash fractions were extracted for the bottlenecks of health centers fractions and each of the themes include three sub categories. Cash fractions are related to the difference between the franchise for total provided services and deposits to the university account and include three sub categories of discounts fractions (Patient deductions), registration fractions, and fractions relating to health centers depositing cash incomes in the university account.

Discounts fractions: These fractions are related to the visit fees which have not been received by providers. According to the opinions of participants, discounts fractions happen in cases such as discounts for colleague and order of the center head. There are no regulations for discounts and thus service providers act based on their own opinions. For example participant no.2 has stated that “… we don’t know which cases are subject to discounts and there is no specific directive in this regard…”.

Registration fractions: These fractions resulted from not registering the provided services and it occurs in cases that the individual directly goes to the providers (thus without paying for franchise). One of the causes of registration fractions is providers’ unawareness.

Fractions for depositing cash incomes: This fraction happens due to incomplete deposit of cash incomes and relates to the difference between the deposited amount to the university account and cash incomes of the center. Participant no.2 said that “… Considering that there is a monthly limit for cash incomes and that the fund system will be disabled after reaching this limit, cashiers will have to deposit the incomes to the university account and activate the system using deposit receipt number. But due to the low security of the system, the system is activated with previous deposit receipt numbers.

Another theme for fraction bottlenecks is non-cash fractions that emphasizes non-payment for non-cash incomes and includes three subcategories of documentations sending fractions, fractions per capita, and insurance deductions.

Fractions related to sending documents: These fractions are related to incomplete sending of documents to insurers. According to the opinions of participants, this happens due to non-delivery of prescriptions from health centers to health networks and from health networks to insurers. Participant no. 11 stated “… When I was checking prescriptions in the month before, I noticed that the numbers of ECGs were registered as 10 but 2 prescriptions were received for them…”.

Insurance deductions: “Insurance deductions” refers to the money not reimbursed by insurers for health services. Based on the experience of the participants, major causes of insurance deductions are related to service provision outside the commitment of insurers and low quality of documentations.

Shortcomings and problems of prescriptions and sent documents to the insurers are other cases of insurance deductions that happen frequently. Significant proportion of the problems in prescriptions is related to the performance of doctors.

Fractions per capita: This item refers to the fractions applied in the family physician program by Iran’s General Office of Health Insurance. It is the difference between family physician per capita and the received income by the health networks and includes adjusted monitoring, quantitative adjustment, per capita adjustment, and population adjustment. Population adjustment is determined according to the number of the population covered by the physician and in cases that the covered population is more than the previously specified number; certain shares are reduced from per capita. Per capita adjustment refers to the lack of settlement of a family physician in a center and quantitative adjustment is related to non-functional days of the providers during the month. Monitoring adjustment also indicates the fractions for monitoring ratings of these centers by the Health Insurance Organization.

According to the opinions of the participants, six themes were extracted for causes of fractions and their solutions.

**These include:** causes and solutions for fractions per capita, insurance deductions, fractions related to sending documents, registration fractions, discounts fractions, and incomplete (partial) deposit of cash incomes.

Lack of physicians was proposed as the most important and highest priority cause for per capita fraction by the participants. Given the definition of population adjustment, lack of an appropriate dedication of population for centers was also proposed along with the lack of physicians as one of the most important causes for fractions per capita. Inappropriate population dedication means defining and determining the population covered by a health center without considering geographical condition and natural behavior of the people of that region.

Causes for health center fractions and their subcategories are presented in Table 2 in detail.

**Table 2:** Causes for health center fractions

Themes for fraction causes	Subcategories
Fractions related to sending documentations	Lack of physicians
	Inappropriate dedication for the covered population
	Excessive referrals
	Incomplete health center documents
	Disregarding other items of monitoring checklist for health insurance organization
Insurance deductions	Unawareness of the physicians regarding instructions
	Inattention of the physicians to incomes and fractions due to the lack of connection between physician incomes and the amount of fractions
	Low skills and knowledge of income section employees for examining prescriptions
Fractions related to sending documentations	Inattention of health center cashiers and inconsistency of the health networks
	Lack of complete examination of the prescriptions and insurance lists in the health networks
Registration fractions	Employing staff with low educational levels as cashiers
	Insufficient training of cashiers
	Providing medical services to people without reception
Discount	Lack of a clear executive bylaw
	Lack of follow up by the networks
Incomplete deposition of cash incomes	low security of fund system

With active cooperation of the participants in this study and based on the identified causes, important and prioritized solutions for reducing fractions were proposed. Solutions for reducing health center fractions and their subcategories are presented in Table 3 in detail.

**Table 3:** Solutions for reducing health center fractions

Themes for solutions of reducing fractions	Subcategory
Solutions for per capita fractions	Supplementation of medical staff by recruiting physicians and employing physicians of the health centers that have two or three doctors as replacement doctors
	Modifying the dedication of population in health centers in which population was defined regardless of natural behavior of the people
	Organizing the system of referrals through clinical education groups
	Extracting causes for monitoring fractions and informing staff
Solutions for insurance deductions	Retrieving list of covered services and medicines from insurers and informing providers
	Retrieving respective instructions from insurers and training physicians
	Giving feedbacks to physicians about the number, causes, and Rial figures of fractions
	Constant evaluation of health centers and payroll deduction based on the fraction amounts
	Training and informing income section staff about instructions at quarterly meetings
Solutions for fractions related to sending documentations	Paying serious attention to cashiers and holding monthly meetings for them in order to keep exchanging opinions and constant training
	Documenting the process of sending documents to insurers and sending the procedure to health networks for acting based on it.
Solutions for registration fractions	Paying serious attention to cashiers and holding monthly meetings for them in order to keep exchanging opinions and constant training
	Preparing instructions and documenting reception procedure and sending them to health centers
Solutions for discounts fractions	Formulating discount bylaws and delivering and implementing it
Solutions for fractions related to incomplete deposit of cash incomes	Using bar code or “Point of Sale” devices based on the conditions of the health center (e.g. in centers with high incomes)

According to the identified solutions, it was proposed to prepare a comprehensive package as “fractions reduction package” such that the prepared package includes all the instructions, bylaws, and documented procedures (flow charts) and requirements of insurers.

## 4. Discussion

The present study was an attempt to identify bottlenecks and causes of fractions of dedicated incomes in health centers and to provide solutions for their reduction. Based on results, these Items are related to all the steps of converting service to income and include the steps of providing services, registering, sending, and receiving incomes.

Based on the opinions of the participants, non-cash fractions are more important and have higher priority than cash fractions and have been emphasized by majority of participants.

For the theme of cash fractions, “discounts fractions” has significant importance and is one of the financial indicators in health services institutions (Janati, Valizadeh, & Asghari-Jafarabadi, 2014). As the results of studies by Chuma (Chuma, Musimbi, Okungu, Goodman & Molyneux, 2009) and Plaetse (Plaetse, Hlatiwayo, Eygen, Meessen & Criel, 2005) showed, discounts for patients in Zimbabwe and Africa had lead to reduction of institutions` revenues and providers were faced with financial problems.

Lack of a clear bylaw for discounts and uncertainty of employees in applying discounts are among the influential factors for discounts fractions that cause employees to treat this regard based on their personal preferences. Based on various directives of Ministry of Health in Iran, like many countries (Chuma et al., 2009; Plaetse et al., 2005; Reed, Graetz, Fung, Newhouse, & Hsu, 2012), providing services to target groups and individuals with specific conditions is free of charge. Therefore, formulating a bylaw for discounts and instructing employees about it and careful supervising on its implementation can be an effective manner for reducing this part of fractions.

Another issue in the category of cash fractions is fractions related to incomplete deposit of cash incomes, which is caused by the poor performance of cashiers and receptionists in health centers. Difficulties of user-fee revenues collection and administration costs are the problems of the management in health centers which have negative effect on the revenue amount and quality improvement (Mubyazi et al., 2006). Lack of control at the level of provincial health networks allows these fractions to be increased.

For the theme of non-cash fractions, sending fractions are of little importance due to low incidence rate and amount. Meanwhile, the other two subcategories (per capita and insurance deductions) are of significant importance and were emphasized by majority of participants.

Challenges related to reimbursement of insurance claims and insurance deductions are the significant problems in health services institutions in Iran, as evidence show that nearly one quarter of medical records of hospitals had at least one type of deductions (Abolhallaje et al., 2013; Tavakoli et al., 2015). Various factors are responsible for incidence of insurance deductions in health care institutions. Because of the similar condition and essence of the actions for insurance deductions in hospitals and health centers, many incidence factors of hospital deductions can be effective for health center deductions. The results for study of “Khademolqorani” (Khademolqorani & Zeinal Hamadani, 2015) indicate, low quality of documents, failure to submit full documents and services and medicines provided outside the commitment of insurers are among the influential factors in the incidence of insurance deductions at hospitals. Low quality of medical records is considered as one of the potential factors in improper performance of medical centers (Esposito, Pelullo, Agozzino, & Attena, 2013).

Given the main role of physicians in insurance deductions, most causes for these fractions are related to the physicians and corrective solutions should also focus on the physicians. Therefore, training and informing physicians with suitable methods and giving feedback to them can become an effective action for making physicians aware and sensitive to the subject of incomes and fractions.

Since implementation of family physician program and its continuation requires resource supplication such as financial resources, understanding the responsible factors for loss of resources can have a great influence on controlling this problem. Per capita fractions is one of the factors responsible for loss of financial resources in family physician program and it is considered as the most common and most important fraction in health centers by participants of this study. Considering the different aspects of per capita fractions, different causes are also responsible for the incidence of these set of fractions. Lack of physicians was selected as the most prioritized cause since it had the major share in the set of these fractions (per capita adjustment, quantitative adjustment, and population adjustment). Lack of physicians is one important challenge of family physician program in Iran which must to be considered significantly (Kalhor, Azmal, Zakariakiaei, Eslamian & Tabatabaee, 2014; Rabinowitz, Diamond, Markham, & Santana, 2011).

Considering the influence of supplying family physicians in health centers on reducing per capita fractions, two solutions were proposed for solving this problem: recruiting new physicians and employing urban health centers physicians (doctors who are outside the family physician program) and centers that have two or three doctors as replacement doctors. Despite having a relatively significant role in solving shortage of doctors, employing doctors who are outside the family physician program is faced with several challenges; In this case, the role of family physician as the health team leader is disrupted and also it is not possible to implement in provinces without urban health centers. Monitoring adjustment is another aspect of per capita fractions and can be studied in eight different aspects including monitoring adjustment in medical services, midwifery, pharmaceutical, laboratory, radiology, dentistry, performance of health centers and provincial health centers. After not complying with predetermined standards and not achieving the complete score in the evaluation by health insurance office, health centers are subject to these fractions, which exceeding referrals and incomplete documentations have the most shares in fractions. It seems the above factors require careful examination for identifying root causes.

Poor referral system and exceeding referrals of patients to higher levels are the important challenges of primary health care in Iran. Various factors such as patient, society, family physicians, specialist doctors and practice characteristics are effective in referral rate. So development of referral system is a necessary order in family physician program (Esmaeili, Hadian, Rashidian, Shariati & Ghaderi, 2015; Nekoei-Moghadam, Sadeghi, & Parva, 2012; ODonnell, 2000). Therefore, a comprehensive perspective is required for designing interventions to solve this cause and all factors should be considered carefully. Considering the significant influence of physicians and specialist doctors, organizing the system of referrals with a focus on specialist doctors and with the cooperation of clinical education groups can play an important role in this regard.

Cashiers inattention or unawareness in different fields can lead to the incidence of significant fractions. Fractions related to sending documents and registration fractions are among these cases. Paying serious attention to cashiers, constant trainings for them, preparing and sending instructions and related procedures and constant monitoring of the performance of health center cashiers can prevent these fractions.

Since samples were selected from one province only, to generalize the findings to target population is a limitation in our study.
